# Immunity and Identity: From the Arterial Wall to the Architecture of Self

**DOI:** 10.31662/jmaj.2025-0541

**Published:** 2026-01-09

**Authors:** Atsushi Mizuno

**Affiliations:** 1 Department of Cardiovascular Medicine, St. Luke’s International Hospital, Tokyo, Japan

**Keywords:** atherosclerosis, immunity, vascular biology, spatial transcriptomics, single cell

Atherosclerosis remains one of the most fundamental chronic inflammatory disorders underlying coronary artery disease, stroke, and peripheral arterial disease. Far from being a mere disorder of lipid deposition, it represents a complex immune-inflammatory response to vascular injury, as first articulated by Ross and colleagues in the *response-to-injury hypothesis* ^(1)^. Decades of research have delineated the sequence of endothelial injury, lipid accumulation, monocyte recruitment, macrophage transformation into foam cells, smooth muscle cell proliferation, and the formation of the fibrous cap.

The tripartite structure of the arterial wall―intima, media, and adventitia―has long served as the architectural basis for mechanistic models of atherosclerosis. Autopsy and animal studies established macrophages and T cells as key drivers of lesion progression, with inflammation acting as the decisive factor in plaque vulnerability. The seminal work of Renu Virmani and colleagues at the CVPath Institute established the morphological concept of the thin-cap fibroatheroma as the prototypical vulnerable plaque, demonstrating that its cellular composition and microstructural architecture determine the plaque’s propensity to rupture ^(2)^.

Within this lineage of discovery, the review by Adachi et al. ^(3)^ at the CVPath Laboratory represents a synthesis of classical pathology and modern molecular cartography. By integrating traditional histopathology with single-cell and spatial technologies, the authors redefine atherosclerosis as a living, multidimensional ecosystem rather than a static lesion. Their review highlights the transformative power of single-cell RNA sequencing, single-cell assay for transposase-accessible chromatin sequencing (scATAC-seq), cellular indexing of transcriptomes and epitopes by sequencing (CITE-seq), T-cell receptor (TCR) sequencing, and spatial transcriptomics in mapping the immune architecture of plaques. Through these approaches, the once-linear model has evolved into a spatially organized immune network, where macrophage and T-cell subsets occupy specialized niches―ranging from inflammatory macrophages in the fibrous cap to TREM2^high^ foam cells near lipid cores. A key insight is that molecular data acquire meaning only within spatial and contextual relationships. Spatial transcriptomics thus provides not just coordinates, but a framework for reconstructing the cellular networks that drive disease. This spatial logic reveals that cell localization and interaction―not sheer abundance―dictate atherosclerotic evolution.

The next frontier in atherosclerosis research is defined by the emergence of highly multiplexed spatial imaging and dynamic network analysis, technologies that hold great promise for uncovering novel mechanisms and guiding the development of future therapies. Co-Detection by Indexing and Multiplexed Ion Beam Imaging enables the simultaneous visualization of more than 40 proteins within a single tissue section, allowing near three-dimensional reconstruction of immune cell interactions and spatial organization ^(4)^. These approaches move beyond static descriptions of plaque architecture to reveal how cells communicate and coordinate across spatial gradients within the vascular microenvironment. Complementary methods such as Perturb-seq, which integrates Clustered Regularly Interspaced Short Palindromic Repeats (CRISPR)-based genetic perturbation with single-cell RNA sequencing, enable causal testing of how specific regulators reshape these cellular networks in real time. The wealth of molecular and cellular data generated through these approaches is now being integrated with artificial intelligence and computational modeling to construct digital twins―virtual representations of patient-specific vascular biology that can simulate plaque evolution and therapeutic response. Together, these converging technologies are expected to transform atherosclerosis research from static observation to predictive, mechanism-based precision medicine.

Adachi et al. ^(3)^ delve deeply into the immunological mechanisms operating at the microscopic level. Immunity represents a dynamic process of continuous negotiation between self and non-self, in which cells constantly redefine the meaning of “self” in response to environmental stimuli and the passage of time. This molecular form of self-recognition resonates with the macrocosm of human existence―our ongoing effort to situate the self within social and environmental contexts. At both scales, the boundaries between self and other are not fixed but evolve through cumulative temporal interactions and mutual influence. Just as the macrophage transcriptome shifts according to its spatial niche, human physiology and behavior fluctuate in cyclical and nonlinear patterns influenced by circadian rhythms, stress, and social environments. These multilayered and temporal oscillations are not merely morphological but extend to physiological responses such as blood pressure variability―woven through time like a living tapestry ^(5)^. Such phenomena underscore the importance of perceiving life not as a linear process but as a hierarchically structured and interdependent system. At both the micro and macro scales, biological and behavioral networks share a common logic―the principle of self-organizing adaptive systems in which local interactions give rise to global order. Across molecules, cells, individuals, and societies, distinct rhythms interfere and overlap, shaping a “dynamic equilibrium” that embodies both scientific precision and philosophical beauty.

From this perspective, the review by Adachi et al. ^(3)^ transcends traditional pathology. It redefines atherosclerosis not as a static disease but as a spatially organized living system, and not merely as a localized vascular event but as a universal model of integrative biology.

## Article Information

### Conflicts of Interest

None

### Disclaimer

Atsushi Mizuno is one of the Editors of *JMA Journal* and on the journal’s Editorial Staff. He was not involved in the editorial evaluation or decision to accept this article for publication at all.
